# Percutaneous needle fasciotomy in Dupuytren contracture: a register-based, observational cohort study on complications in 3,331 treated fingers in 2,257 patients

**DOI:** 10.1080/17453674.2020.1726057

**Published:** 2020-02-14

**Authors:** Laura Houstrup Therkelsen, Simon Toftgaard Skov, Malene Laursen, Jeppe Lange

**Affiliations:** a Elective Surgery Centre, Silkeborg Regional Hospital, Denmark;; b Department of Orthopaedic Surgery, Horsens Regional Hospital, Denmark

## Abstract

Background and purpose — Percutaneous needle fasciotomy (PNF) is a minimally invasive treatment option for Dupuytren contracture, which has gained momentum worldwide in recent years. However, evidence regarding safety and severe complications associated with PNF is sparse. Thus, we evaluated safety of a specific PNF method in the largest cohort reported in literature.

Patients and methods — This is a single-center, register-based, observational study on PNF treatments between 2007 and 2015. The study cohort was identified by the Danish National Patient Registry, and diagnosis codes and procedure codes were used to identify potential severe postoperative complications such as: tendon rupture, nerve damage, infection, amputation, and reflex dystrophy for all index treatments. The Danish National Prescription Registry was used to identify non-hospital-treated infections. All index treatments and postoperative complications were verified by review of medical records.

Results — 2,257 patients received PNF treatment of 3,331 treated finger rays. Median follow-up was 7.2 years (interquartile range: 4.9–9.5 years, range 1–12 years). 4 patients sustained flexor tendon rupture. 1 patient had digital nerve damage. 2 patients had an infection treated in hospital, while 31 patients received antibiotics in the primary sector for an infection or based on suspicion of an infection after PNF. None of the infections required surgical intervention. No finger amputations or ipsilateral upper limb reflex dystrophy cases were registered in relation to the procedure.

Interpretation — Percutaneous needle fasciotomy for Dupuytren contracture is a safe procedure with a low rate of severe postoperative complications when a specific PNF method is applied.

Dupuytren contracture is a benign fibroproliferative disease that affects the aponeurotic fibers in the palm, causing permanent flexion contracture of the fingers. This extension deficit may imply disability in activities of daily living for the affected patients (Wilburn et al. 2013). There is no cure for Dupuytren contracture, but several symptomatic treatment options exist. However, there is no consensus as to the optimal treatment in individual cases (McMillan et al. 2017).

Percutaneous needle fasciotomy (PNF) is performed as a minimally invasive surgical procedure under local anesthesia in an outpatient setting. A fine syringe needle is used to perforate the Dupuytren cords repeatedly until the finger can be extended. The PNF technique was reintroduced in the late 1970s by a group of French rheumatologists, and has been performed in Denmark at our center since 2006, according to the method of Lermusiaux and Debeyre (1979). 2-year results after the initial introduction of PNF treatment were promising (Rahr et al. 2011).

Several randomized controlled trials have shown that PNF is not inferior to collagenase clostridium histolyticum (CCH) injection regarding complication rate and clinical outcome (Scherman et al. 2016, Skov et al. 2017, Strömberg et al. 2016, 2018). Even though PNF has shown reliable clinical results, evidence regarding complications associated with PNF is sparse. A potential risk of iatrogenic nerve and tendon damage during the procedure has been stated as a specific concern.

We evaluated the safety and severe complications following the PNF method applied in our institution.

## Patients and methods

### Study design

This was a single-center, register-based follow-up study with data validation by review of medical records.

### Study cohort

The study cohort was established by identifying all patients receiving PNF treatment for Dupuytren contracture at Silkeborg Regional Hospital, Denmark, between January 1, 2007, and December 31, 2015 in the Danish National Patient Registry (DNPR) (data extraction specified in Table 1, see Supplementary data). 2007 was chosen as it was the first full year of PNF treatment in our department. The index procedure was defined as the patient’s first PNF treatment during the study period. Thereby, any individual patient is only represented once.

**Table 5.** Distribution of treated hand, finger, and joint affection

Factor	Number
Affected hand	
right	1,723
left	1,592
NS	16
Affected finger	
little finger	1,445
ring finger	1,265
middle finger	408
index finger	92
thumb	88
NS	33
Affected joint	
metacarpophalangeal joint	2,696
proximal interphalangeal joint	1,713
distal interphalangeal joint	144
NS	164

NS: Not specified in medical record.

The sum of treated hands and fingers (n = 3,331) exceeds the number of patients (n = 2,257) as some patients had several fingers treated in same session. Likewise, the sum of affected joints (n = 4,717) exceeds the number of treated fingers as more joints on the same finger were affected.

### Percutaneous needle fasciotomy procedure: the Silkeborg method

Percutaneous needle fasciotomy (PNF) is performed in an outpatient setting. Following the standard disinfection procedure and sterile draping, 0.1–0.2 mL of 1% lidocaine with epinephrine is injected into chosen sites of the Dupuytren cord, using a small, 25-gauge syringe needle. The anesthetics is injected only intradermally to maintain full sensibility of the PNF-treated finger during the entire procedure. This specific procedural monitoring is believed essential to minimize the risk of nerve affection. If the patient experiences any kind of nerve affection following the injection of the local anesthetics before the procedure begins, or any nerve sensation during the procedure, the procedure is stopped, and patients with unreleased contractures are offered a repeated procedure another day. This concept of nerve protection was meticulously adhered to in all treated patients in this cohort, and we believe it of utmost importance when performing PNF.

The technique, in which the cord is then weakened, is a combination of repeated needle-tip perforations into the cord, and cautious pendulum cutting of the cord, with a 25-gauge needle at a slow pace, and simultaneously passively stretching the finger to rupture the Dupuytren cord. To protect against nerve affection also during the procedure a small 25-gauge needle is always used. Several needles may be used, if dull. When the Dupuytren cord is sufficiently weakened, the finger can be stretched manually and any potential residual cord strings can be slowly, but forcefully ruptured. Additional local anesthetic may be required to achieve a final manipulation at the end of the treatment, but only after the perforation/cutting has definitely ended. The procedure is typically applied at 1–5 different sites, including Dupuytren cords affecting both metacarpophalangeal and proximal interphalangeal joints, depending on the severity of the contracture and the number of finger rays to be treated (Rahr et al. 2011, Skov et al. 2017).

The PNF procedures were performed by 4 orthopedic surgeons, 1 PNF-trained resident doctor, and 1 PNF-trained registered nurse.

### Data collection

All identified procedures were verified by review of medical records to ensure that the index procedures were PNF. Simultaneously, baseline information on treated hand, finger, and joint affection, and any special perioperative circumstances, was collected from the medical records. Patients who died within 1 year after the index procedure were excluded due to the insufficient follow-up time. Likewise, we excluded patients residing abroad due to inability to follow-up.

After establishing the study cohort, information on succeeding diagnosis codes and procedure codes relative to the index procedure was extracted from the DNPR to locate potential postoperative complications for a retrospective review in the medical records (data-extraction strategy specified in Tables 2 and 3, see Supplementary data).

The dataset was time limited, assuming that complications such as flexor tendon ruptures, nerve damage, and amputations would present within 1 year, infections within 1 month, lesions within 3 months, and reflex dystrophy within 5 years from index procedure.

The dataset was compiled on September 20, 2018.

Presumably, all operations and severe complications are registered in the DNPR, because reimbursement for both government hospitals and private clinics relies on registration (Lynge et al. 2011). However, the primary sector is not required to report to DNPR. In order to avoid this information bias regarding the postoperative infection rate, we extracted data on anatomical therapeutic chemical (ATC) codes from the Danish National Prescription Registry, which holds information on prescribed antibiotics (specified in Supplementary data, Table 4). Thereby, all infections were most likely accounted for, covering the full spectrum from minor superficial infections to severe deep infections. The indications for prescribed antibiotics were audited nationwide in the medical records. If the information was unavailable in the hospital medical records, we contacted the patient’s general practitioner.

Follow-up time was determined as time from index procedure to either date of data extract or date of death. Time of death and information on country of residence were extracted from the Danish Civil Registration System (Pedersen 2011).

### Statistics

Data extracts from the National Health Administrative Registries were available on the Research Engine administrated by the Research Services of the Danish Health Data Authority. (Data management and statistical analysis were performed in the open source RStudio; R Foundation for Statistical Computing, Vienna, Austria, version 1.1.463.)

### Registration, funding, and conflicts of interest

Study approvals were obtained from the Danish Patient Safety Authority (3-3013-2261/1) for access to review of medical records and from the Danish Data Protection Agency (1-16-02-711-17). According to Danish law, no further approvals were required. Data from medical records were collected under current regional data protection legislature, with acceptance from the Head of Department of the relevant departments or the patient’s general practitioner. The study was conducted in accordance with the Law of Processing Personal Data and the Danish Health Act.

Funding was received from Rieck-Andersens Familiefond Aarhus, the Danish Rheumatism Association, and Elisabeth & Karl-Ejnar Nis-Hanssens Mindelegat.

All authors declare no conflict of interest.

## Results

2,308 patients were identified in the DNPR. All index PNF treatments were verified by review of medical records; 28 patients did not receive PNF treatment, 21 patients died within the first year after index procedure, and 2 patients had residence abroad.

The study cohort included 2,257 patients with 3,331 treated finger rays. 1,777 (79%) were men. Mean age at index was 66 years (18–96). Median follow-up was 7.2 years (IQR 4.9–9.5, range 1–12 years). The distributions of treated hand, finger, and joint affection are shown in Table 5.

### Complications (Figure)

110 of the 2,257 treated patients were identified to have a complication potentially related to the PNF treatment. 4 patients had flexor tendon rupture after PNF, double flexor tendon rupture (both superficial and deep flexor tendons) occurred in 3 cases, while 1 patient had only the profound flexor tendon ruptured. In the 3 cases of double tendon injury, the patients had tenorrhaphies subsequently. The double flexor tendon ruptures were registered 7, 8, and 9 days postoperative, respectively. The patient who ruptured the deep flexor tendon reported that it occurred 3 days after PNF and was treated nonoperatively. 1 patient sustained digital nerve damage, while 5 patients had temporary nerve symptoms that resolved within 2 weeks after the index procedure. None of the patients with transient or persisting nerve symptoms received further treatment, and no later contacts were registered regarding nerve laceration.

2 cases of infections were identified via the DNPR nationwide data. These patients were seen in the outpatient clinic, and received oral antibiotic treatment and simple wound care.

75 patients were registered in the Danish National Prescription Registry as receiving antibiotics within 1 month after PNF (including the above-mentioned 2 patients with postoperative infections). 42 patients received antibiotic treatment for other reasons, and 18 had antibiotics prescribed in general practice to treat or to prevent an infection following the PNF treatment. In 13 cases, there was no documentation available in the hospital records or in the general practitioner’s records specifying the indication for the antibiotic treatment. None of the infections required intravenously administered antibiotics or surgical treatment.

4 patients contacted our department after the index procedure with a skin rupture needing the bandage replacing but without signs of infection.

In treatment of 70 finger rays (in 63 patients), the PNF procedure was discontinued due to paresthesia or accidental skin lesion. Unreleased contractures were offered a new procedure another day, but no further wound complication or nerve affection in relation to these cases were documented in the medical records.

No complications led to amputation. However, 3 patients had the digit of the index procedure amputated within the first year due to insufficient effect of the PNF treatment. This was not defined as a complication. No ipsilateral upper limb reflex dystrophy cases were registered in relation to the procedure.

## Discussion

In this register-based follow-up study on 2,257 patients, we found low rates of severe postoperative complications including flexor tendon rupture (n = 4), nerve damage (n = 1), and infection (n = 33) following our PNF method.

One of the key findings in this study is the extremely low prevalence of nerve damage identified. We acknowledge the risk of information bias due to insufficient registration, or that patients with a nerve affection are simply not seen by a doctor for various reasons and the consequences this would have for our possibility of identifying these patients. However, we truly believe our result to be genuine regarding the low prevalence of nerve affection, when the described PNF technique is applied. This is supported by the results in our randomized controlled trial (Skov et al. 2017), and also in other randomized controlled trials involving a similar PNF procedure (Scherman et al. 2016, Strömberg et al. 2016). It is important to highlight a methodological aspect of the PNF procedures performed in this cohort: PNF is performed using a fine needle (25G), at a slow pace, and under low doses of local anesthetic allowing the full sensibility of the finger to be monitored during the procedure, to minimize the risk of digital nerve damage. We believe this is reflected in the low rate of nerve injury (Skov et al. 2017). In addition, the procedures were performed by only 6 individuals during the study period, thus a certain expertise is represented, and they were all specially trained in the PNF method.

In 2018, a review stated that PNF has significantly lower complication rates compared with open fasciectomy and CCH injection (Elzinga and Morhart 2018). In 2017, a systematic review of 113 studies assessing the incidence of complications associated with different treatment options for Dupuytren contracture found a pooled complication rate of 19% after PNF (Krefter et al. 2017). They included both major and minor complications, and found that the highest prevalence of nerve and vessel lesions occurred following open surgery. This review did not consider the severity of the complications, and raised the question as to whether a skin tear, which can heal without further intervention, should be considered a complication. Our study is based on Danish administrative registries, merely investigating complications requiring contact with the healthcare system. Consequently, minor skin tears will not be registered.

A single-center retrospective study on open surgeries for Dupuytren contracture performed between 1956 and 2006 at Erlangen University Hospital, Germany, was published in 2007 (Loos et al. 2007). The study included 2,919 patients, corresponding to the number of patients in our study (2,257). Nerve injuries were observed in 108 cases (3.7%), tendon injuries occurred in 4 cases (0.2%), skin necrosis was documented in 2.6% of all operations, and wound infection was observed in 94 patients (3.2%). The reported tendon injury risk after open surgery is similar to our findings after PNF (0.18%). But we found remarkably lower rates of nerve injuries (0.04%) and infections (1.5%). Also, we did not identify any cases of skin necrosis. However, the complication rate reported by Loos et al. may be underestimated as the authors looked only at data from their own local medical records. In contrast, we included nationwide data on potential PNF complications registered by other hospitals/departments.

Comparing different treatment modalities for Dupuytren contracture based on available literature is a cumbersome task due to variations in follow-up time, different definitions of complications, and noncomparable study cohorts. However, by reviewing the literature (Table 6, see Supplementary data) in combination with our results, PNF appears to be a safe treatment modality for Dupuytren contracture. Dupuytren contracture is relatively common, and it is noticeable that the number of studies on PNF is limited, and many of them are small case series.

In 13 of our cases, in which antibiotics were prescribed, there was no documented indication available in the hospital records or in the general practitioner’s records. In those cases, and with the intention of avoiding underestimation of the risk of infection following PNF treatment, we assumed that antibiotic prescription was associated with PNF treatment, leaving the total infection rate at 1.5%.

Complex regional pain syndrome (CRPS) is known to be a complication associated with Dupuytren contracture interventions (Krefter et al. 2017). 1 patient in our cohort was registered with CRPS (ICD-10: DM89.0) almost 4 years after index procedure, but this was found to be related to a distal radius fracture and not the PNF treatment. We examined CRPS cases up to 5 years after the index procedure and included differential diagnoses, but found no other CRPS cases. Of course, there is a risk of patients being misdiagnosed or not diagnosed at all. However, if a patient in Denmark experiences severe symptoms resembling CRPS, we believe they will most likely be referred to a hospital clinic for an evaluation, and thereby become adequately registered.

### Study limitations and strengths

The major strengths of this study are the size and complete nationwide follow-up, combined with data verification by manual review of medical records. However, there are limitations to this study. (1) This is a retrospective register-based study, and the complications following PNF were identified through the DNPR and the Danish National Prescription Registry. The data were therefore not prospectively targeted for this study. (2) Complications were identified based on a data extract of diagnosis codes, procedure codes, and ATC codes. Thus, complications reported with codes not included in our dataset will not be identified. (3) There is a risk of underestimating complications following PNF if they are inadequately registered, or if patients with potential complication are not seen in hospital clinics or referred by the general practitioner, e.g., nerve affection with low demand in daily activities. Regarding infections, we extracted data covering all infections including patients treated in the primary sector. (4) In contrast to other studies, we did not report minor complications such as skin ruptures. Neither recurrence nor subsequently repeated treatment were regarded as complications. However, when we compare detailed results regarding severe complications, our cumulative complication rate of 1.7% seems similar to those other studies. Moreover, it is noteworthy that no skin lesions led to further wound complication, than could be managed by oral antibiotics and simple wound care.

To our knowledge, this study represents the largest cohort with complete nationwide follow-up regarding safety and severe complications after PNF treatment in the literature. Percutaneous needle fasciotomy for Dupuytren contracture is a safe procedure with a low rate of severe postoperative complications when an appropriate PNF technique is applied (Skov et al. 2017). The importance of this study is further emphasized, as PNF is less expensive than all treatment alternatives for Dupuytren contracture, and it has shown to be cost-effective (Baltzer and Binhammer 2013, Chen et al. 2011).

## Supplementary Material

Supplemental Material

## Supplementary data

Tables 1–4 and 6 are available as supplementary data in the online version of this article, http://dx.doi.org/10.1080/ 17453674.2020.1726057
