# Supplementary material for: Percutaneous needle fasciotomy in Dupuytren contracture: a register-based, observational cohort study on complications in 3,331 treated fingers in 2,257 patients
Source: Acta Orthop. 2020 Feb 14;91(3):326–30. doi: 10.1080/17453674.2020.1726057 (PMC8023958; doi:10.1080/17453674.2020.1726057)
Supplement: Supplemental Material [file IORT_A_1726057_SM6475.pdf]

Supplementary data

Table 1. Study cohort

Code	Factor
DM720:	Palmar fascial fibromatosis (Dupuytren)
KND M09x:	Fasciotomy to the wrist or hand
7002 06x (→ date 03.11.2012) and 6630 32x (date 03.11.2012 →):	Elective Surgery Centre, Silkeborg Regional Hospital, Denmark
2007–2015:	Study period

The study cohort was identified in the Danish National Patient Registry by extract of following combination of SKS codes in the Danish Healthcare Classification System.

Table 2. Succeeding diagnosis codes

Code	Diagnosis
DA46x:	Erysipelas
DA49x:	Bacterial infection of unspecified site
DB95x:	Streptococcus and staphylococcus as the cause of diseases
DB99x:	Other infectious diseases
DG54.6:	Phantom limb syndrome with pain
DG54.7:	Phantom limb syndrome without pain
DG54.8:	Other nerve-root and plexus disorders
DG56x:	Mononeuropathies of upper limb
DG59x:	Mononeuropathies, non-specific
DG64.9:	Other disorders of the peripheral nervous system
DL02x:	Abscess in skin
DL03x:	Phlegmon
DL04x:	Acute lymphadenitis
DM65x–DM68x:	Disorders of synovium and tendon
DM72x:	Fibroblastic disorders
DM79x:	Other soft tissue rheumatism
DM89.0:	Reflex dystrophy
DS6xx:	Injuries to the wrist and hand
DT80x:	Complications following injections
DT81x:	Complications of procedures
DT88x:	Other complications of surgical and medical care
DT89x:	Infections caused by hospital treatment

Succeeding nationwide diagnosis codes were extracted from the Danish National Patient Registry to identify postoperative complications following PNF. The diagnosis codes are confined from each patient's admission into the study cohort (index date) until the date of data extract (September 20, 2018).

Table 3. Preceding and succeeding operation codes

Code	Diagnosis
KND xxx:	Procedures to the wrist or hand

Succeeding nationwide operation codes extracted from the Danish National Patient Registry were used to identify postoperative complications requiring further surgery.

Table 4. Anatomical therapeutic chemical (ATC) codes

Code	Antibiotic type
MJ01:	Antibiotics for systemic use
MJ01CE02:	Phenoxymethylpenicillin
MJ01CF01:	Dicloxacillin
MJ01CF05:	Flucloxacillin
MJ01CR02:	Amoxicillin with clavulanic acid
MJ01CA04:	Amoxicillin
MJ01CA01:	Ampicillin
MJ01FA*:	Macrolides, e.g., erythromycin, roxithromycin, clarithromycin, azithromycin

Data extracted from the Danish National Prescription Registry were used to identify infections treated at general practices or by emergency doctors. The data contain information concerning prescribed antibiotics on all included patients within a month after the index PNF procedure.

Table 6. Review of reported complications after PNF in literature

Author (year)	Study design [a]	No. of patients (finger rays)	Follow-up, mean (range)	Complications
Strömberg et al. (2016, 2018) [1,2]	RCT	60 (60)	2 years	Skin rupture (38% of the patients)
Skov et al. (2017)3	RCT	19 (19)	2 years	Wound complication (5% of the patients)<br>Skin rupture (10% of the patients)<br>Local discomfort (24% of the patients)
van Rijssen et al. (2006, 2012) [4,5]	RCT	52	5 years	Skin rupture (48% of the procedures)<br>Paresthesia (7% of the procedures)
Scherman et al. (2016) [6]	RCT	38 (42)	1 year	Skin rupture (8% of the patients)<br>Infection treated with drainage and oral antibiotics (2% of the patients)<br>Sporadic pain or discomfort with activity at 1 year (10% of the patients)
Molenkamp et al. (2017) [7]	R	451 (470)	Not available	Permanent sensory loss (0.2% of the patients)<br>Tendon rupture requiring surgery (0.2% of the patients)<br>Temporary paresthesia (1.3% of the fingers)<br>Superficial infection treated with antibiotics (1.1% of the procedures)<br>Skin tear (9.1% of the procedures)
Pess et al. (2012) [8]	R	431	3 years<br>(3–6.2)	Nerve laceration (0.1% of the fingers)<br>Temporary neuropraxia (1.2% of the patients)<br>Skin tear (3.4% of the procedures)
Foucher and Medina (2003) [9]	P	100	3.2 years	Permanent digital nerve damage (1% of the patients)<br>Paresthesia (1.3% of the procedures)<br>Tinel's sign (0.6% of the procedures)
Rahr et al. (2011) [10]	R	92 (130)	2 years	Superficial infection (1.1% of the patients)<br>Temporary nerve symptoms (8.7% of the patients)
Moog et al. (2019) [11]	R<br>R	53 (68)	31 months<br>(12–50)	Transient dysesthesia (1.8%)<br>Skin tear (3.8% of the procedures)
Abe and Tokunaga (2015) [12]	P	51 (103)	1 year	Nerve damage (2% of the patients)<br>Temporary neuropraxia (11.8% of the patients)<br>Skin tear (17.5% of the fingers)
Zhou et al. (2016) [13]	R	78	10 weeks<br>(6–12)	Nerve laceration (1.3% of the patients)<br>Uncorrectable contracture (1.3% of the patients)<br>Neuropraxia (2.6% of the patients)<br>Skin rupture (2.6% of the patients)
Mansha et al. (2017) [14]	R<br>R	46 (51)	15 months<br>(5–36)	No complications
Pereira et al. (2012) [15]	R	36 (44)	28 months<br>(12–63)	Skin rupture (16% of the finger rays)<br>Transient paresthesia (9% of the finger rays)
Nydick et al. (2013) [16]	R	30	6 months<br>(3–28)	Edema (17% of the patients)<br>Ecchymosis (10% of the patients)<br>Skin tear (50% of the patients)
Karakaplan et al. (2019) [17]	R	28 (75)	29 months<br>(12–60)	Superficial infection treated with oral antibiotic (3.6% of the patients)<br>Skin rupture treated with suture (25% of the patients)<br>Skin rupture treated with skin graft (7% of the patients)<br>Hypoesthesia in the ulnar digital nerve (3.6% of the patients)
Cheng et al. (2008) [18]	R	8 (13)	22 months<br>(3–45)	Skin tear (27% of the treated points)

[a] RCT = Randomized controlled trial, R = Retrospective, P = Prospective

References to Table 6:

1. Strömberg J, Ibsen-Sörensen A, Fridén J. Comparison of treatment outcome after collagenase and needle fasciotomy for Dupuytren contracture: a randomized, single-blinded, clinical trial with a 1-year follow-up. J Hand Surg Am 2016; 41(9): 873-80. doi:10.1016/j.jhsa.2016.06.014
2. Strömberg J, Sörensen AI, Fridén J. Percutaneous needle fasciotomy versus collagenase treatment for Dupuytren contracture: A randomized controlled trial with a two-year follow-up. J Bone Joint Surg Am 2018; 100-A(13): 1079-86. doi:10.2106/JBJS.17.01128
3. Skov S T, Bisgaard T, Søndergaard P, Lange J. Injectable collagenase versus percutaneous needle fasciotomy for Dupuytren contracture in proximal interphalangeal joints: a randomized controlled trial. J Hand Surg Am 2017; 42(5): 321-8.e3. doi:10.1016/j.jhsa.2017.03.003
4. van Rijssen A L, Ter Linden H, Werker P M N. Five-year results of a randomized clinical trial on treatment in Dupuytren's disease: percutaneous needle fasciotomy versus limited fasciectomy. Plast Reconstr Surg 2012; 129(2): 469-77. doi:10.1097/PRS.0b013e31823aea95
5. van Rijssen A L, Werker P M N. Percutaneous needle fasciotomy in Dupuytren's disease. J Hand Surg Am 2006; 31(5): 498-501. doi:10.1016/j.jhsb.2006.03.174
6. Scherman P, Jenmalm P, Dahlin L B. One-year results of needle fasciotomy and collagenase injection in treatment of Dupuytren's contracture: a two-centre prospective randomized clinical trial. J Hand Surg Eur 2016; 41(6): 577-82. doi:10.1177/1753193415617385
7. Molenkamp S, Schouten T A M, Broekstra D C, Werker P M N, Moolenburgh J D. Early postoperative results of percutaneous needle fasciotomy in 451 patients with Dupuytren disease. Plast Reconstr Surg 2017; 139(6): 1415-21. doi:10.1097/PRS.0000000000003357
8. Pess G M, Pess R M, Pess R A. Results of needle aponeurotomy for Dupuytren contracture in over 1,000 fingers. J Hand Surg Am 2012; 37(4): 651-6. doi:10.1016/j.jhsa.2012.01.029
9. Foucher G, Medina J. Percutaneous needle aponeurotomy: complications and results. J Hand Surg Brit Eur 2003; 28B(5): 247-31.
10. Rahr L, Søndergaard P, Bisgaard T, Baad-Hansen T. Percutaneous needle fasciotomy for primary Dupuytren's contracture. J Hand Surg Eur 2011; 36(7): 548-52. doi:10.1177/1753193411407245
11. Moog P, Buchner L, Cerny M K, Schmauss D, Megerle K, Erne H. Analysis of recurrence and complications after percutaneous needle fasciotomy in Dupuytren's disease. Arch Orthop Trauma Surg 2019. doi:10.1007/s00402-019-03247-y
12. Abe Y, Tokunaga S. Clinical results of percutaneous needle fasciotomy for Dupuytren's disease in Japanese patients. Plast Reconstr Surg - Glob Open 2015; 3(5) :1-8. doi:10.1097/GOX.0000000000000338
13. Zhou C, Selles R W, Slijper H P, et al. Comparative effectiveness of percutaneous needle aponeurotomy and limited fasciectomy for Dupuytren's contracture: a multicenter observational study. Plast Reconstr Surg 2016; 138(4): 837-46. doi:10.1097/PRS.0000000000002560
14. Mansha M, Flynn D, Stothard J. Safety and effectiveness of percutaneous needle fasciotomy for Dupuytren's disease in the palm. J Hand Microsurg 2017; 09(03): 115-19. doi:10.1055/s-0037-1605387
15. Pereira A, Massada M, Sousa R, Silva C, Trigueiros M, Lemos R. Percutaneous needle fasciotomy in Dupuytren's contracture: is it a viable technique? Acta Orthop Belg 2012; 78(1): 30-4.
16. Nydick J A, Olliff B W, Garcia M J, Hess A V, Stone J D. A comparison of percutaneous needle fasciotomy and collagenase injection for Dupuytren disease. J Hand Surg Am 2013; 38(12): 2377-80. doi:10.1016/j.jhsa.2013.08.096
17. Karakaplan M, Ertem K, Polat H, Şah Sakçi M, Oklu Y. Percutaneous needle fasciotomy for the treatment of Dupuytren's contracture. Eklem Hast Cerrahisi 2019; 30(1): 53-60. doi: 10.6506/ehc.2019.58854.
18. Cheng H S, Hung L K, Tse W L, Ho P C. Needle aponeurotomy for Dupuytren's contracture. J Orthop Sur. 2008; 16(1): 88-90.
